# Feasibility of a progressive protocol of high-intensity interval training for overweight/obese, sedentary African American women: a retrospective analysis

**DOI:** 10.1186/s13102-020-00207-7

**Published:** 2020-09-21

**Authors:** Avigdor D. Arad, Jeanine B. Albu, Fred J. DiMenna

**Affiliations:** Division of Endocrinology, Diabetes and Bone, Department of Medicine, Icahn School of Medicine at Mount Sinai, New York, NY USA

**Keywords:** High-intensity interval training, Feasibility, African American women, Overweight/obesity, Exercise adherence, Prescribing intensity

## Abstract

**Background:**

African American (AA) women have a higher prevalence of obesity and related metabolic dysfunction and lower level of physical activity compared to white counterparts. Determining feasible exercise alternatives for AA women is, therefore, paramount. Time-efficient high-intensity interval training (HIIT) might be particularly suited for AA women who cite time constraints as a frequent barrier to exercise adherence. The purpose of this study was to assess the feasibility of a 14-week progressive HIIT protocol for previously-sedentary overweight/obese AA women.

**Methods:**

Twenty-eight healthy, premenopausal (age, 20–40 yr), sedentary, nondiabetic, overweight/obese AA women volunteered to participate in the randomized controlled clinical trial from which these data were retrospectively analysed. After assessment, participants were randomly allocated to a HIIT group (*n* = 14) or a no-exercise control group. The HIIT intervention consisted of 24-min sessions performed three times per week for 14 weeks during which work-interval intensity (75 to 90% of heart rate reserve; HRR) and duration (30 to 60 s) and work/recovery ratio (1:7 to 1:3) were progressed in four stages. Feasibility was assessed based on adherence (attrition rate), perceptual response (RPE) and success rate, which was calculated based on the degree to which target intensities for work intervals were achieved/maintained.

**Results:**

Five of 14 participants (35%) in the HIIT group dropped out during the intervention. One-way repeated-measures ANOVA revealed a significant difference across stages for success rate (*p* = 0.018) with post-hoc analysis indicating a significant difference between stage 1 and the other stages and stage 4 and the other stages. There was no significant difference in RPE across stages (*p* = 0.057).

**Conclusion:**

Albeit based on a limited number of participants, we found an attrition rate that was higher than what has been reported previously for HIIT (~ 17.6%) when previously-sedentary overweight/obese AA women performed a protocol with work-interval intensity progressed from 75 to 90% HRR during a 14-week intervention. With respect to intensity, the precipitous drop for achievement of the target HR during the fourth stage (weeks 8–14) for those who did complete the protocol implies that it might be advisable to restrict work-interval intensity to < 90% HRR.

**Trial registration:**

ClinicalTrials.gov. (NCT04293367). Registered 03 March 2020 – Retrospectively registered.

## Background

High-intensity interval training (HIIT) comprising periods of high-intensity ‘work intervals’ interspersed with recovery intervals of low-intensity exercise or rest has long been a staple of athletic training [1]. However, in recent years, the efficacy and safety of HIIT for non-athletic populations and even for individuals with chronic disease have been explored and results have been promising [2]. In addition to providing a potent stimulus for central and peripheral physiological adaptations that match or exceed those which are induced by traditional continuous endurance training [3], the reduced time commitment required for HIIT might be of particular benefit for individuals unaccustomed to exercise because insufficient time is one of the most frequently-cited reasons for lack of adherence to an exercise program [4, 5]. However, while attractive as speculation, empirical evidence supporting this contention is equivocal. Perri et al. found that for sedentary adults, increasing frequency provided a way to increase the accumulation of exercise without a decline in adherence whereas increasing intensity did not [6]. Moreover, Lunt et al. reported that for overweight, inactive adults at high risk for cardio-metabolic disease, the adherence rate to two different HIIT protocols was relatively low when exercise was performed as part of a group-based activity program (i.e., in a ‘real-world setting’) [7]. Conversely, Vella et al. showed that HIIT allowed for a similar rate of adherence compared to continuous exercise when prescribed for overweight and obese adults [8]. Interestingly, this study involved 3 weeks of supervised training followed by 5 weeks of unsupervised training during which adherence did not decrease for either group [8]. This refutes speculation by Lunt et al. that the physiological adaptations induced by HIIT in the laboratory setting might not reflect what can be achieved in the real world where dropout is more likely [7]. Finally, studies that have examined the affective responses during a HIIT session have also returned equivocal findings [9–11]. The reason(s) for this lack of coherence regarding how well HIIT is physically and/or psychologically tolerated by individuals unaccustomed to exercise is unclear, but might represent different HIIT protocols that were investigated and/or different populations that were studied. With respect to the former, variables that might be particularly important to consider are the intensity at which HIIT work intervals are performed and the volume of the HIIT protocol [12].

By definition, HIIT can comprise work intervals at any intensity that exceeds the maximal sustainable pace; hence, a range of work rates spanning from the ‘critical power’ that demarcates the lower boundary of the severe-intensity domain through peak power to all-out effort (e.g., sprint interval training; SIT) fall under this umbrella term. Furthermore, work-interval intensity is one of the variables that can be increased to provide a progressive overload over the course of an initial HIIT intervention. Generally speaking, HIIT work intervals are performed at an intensity that elicits ≥80% and, in most cases, 85–95% of maximal heart rate [3]; hence, a progressive scheme that spans this range might, in theory, be an applicable one during the initial stage of HIIT. In support of this possibility, in a recent systematic review and meta-analysis, Reljic et al. found that training volume predicted dropout rate for sedentary individuals performing HIIT whereas exercise intensity did not [12]. This would support the belief that individuals who are unaccustomed to exercise should be able to tolerate both psychologically (e.g., maintain adherence to) and physically (e.g., achieve the intensity requirements of) such a progressive HIIT protocol during the initial stage of training as long as volume is not excessive [12].

As previously mentioned, perceived lack of time is a commonly-cited barrier to exercise for the majority of populations across cultures and age span [4, 5]. However, some populations are less likely to exercise than others. For example, compared to their white counterparts, African American (AA) women are less physically active with time constraints specifically cited most frequently as the reason [13]. African American women also possess lower cardiorespiratory fitness (e.g., maximal rate of oxygen uptake and lactate threshold) [14, 15] and a higher prevalence of obesity [16] and insulin resistance [17] compared to their white counterparts The lower oxidative capacity demonstrated by AA individuals appears to be linked to an increased percentage of type II muscle fibers [18], which are serially recruited during physical activity based on intensity of effort [19]. Interestingly, it was recently shown that 6 weeks of HIIT performed by previously-sedentary women resulted in increases in capillary density and mitochondrial content in both principal fibre types [20]. This contrasts lower-intensity sustained endurance exercise which results in serial activation of and, as a result, adaptive stimulation in type I fibres only. Collectively, these differences imply that the time-efficient, effective and, perhaps most importantly, intensity-driven nature of HIIT make it a particularly appropriate exercise alternative to consider for AA women. In this regard, we have found that compared to a no-exercise control group, HIIT improved gas-exchange (as a proxy for lactate) threshold, exercise tolerance and substrate use during incremental leg-cycling exercise for previously-sedentary, premenopausal, nondiabetic, overweight/obese AA women [21]. More recently, Hornbuckle et al. found that for young overweight/obese AA women, HIIT was more effective than continuous exercise for reducing waist circumference and increasing daily steps [22]. However, in that study, dropout rate for the HIIT group (31%) [22] was higher than that which is typically reported for sedentary individuals performing HIIT (~ 17.6%) [12]. Further investigation of the specific characteristics of a HIIT program that might influence the degree to which overweight/obese AA women adhere to and experience success with this type of exercise training might, therefore, be of interest.

The purpose of this study was to re-examine the data that we previously collected and reported on [21] in order to assess the feasibility of HIIT (and, specifically, a progressive protocol that we employed over the course of a 14-week intervention) for previously-sedentary overweight/obese AA women. Specifically we now report on the calculated degree to which the ‘target intensity’ (as indicated by heart-rate response) we had established for the work intervals that were performed during each of the four progressive stages of our HIIT program could be successfully achieved/maintained by the overweight/obese AA women who participated in the study. We also examined perceptual responses (Borg RPE scale; 6–20) to these work intervals. Based on previous reports that: 1.) a range of 85–95% of maximal heart rate is appropriate for HIIT [3]; and 2.) volume predicts dropout rate for sedentary individuals performing HIIT whereas intensity does not [12], we hypothesised that the percentage of successful work-interval sessions completed with intensity progressed from 75 to 90% of HRR would not differ significantly across the four stages of the intervention period thereby indicating that increasing intensity through this range was a viable way to provide a progressive overload when prescribing HIIT for previously-sedentary overweight/obese AA women during the initial 14 weeks of training.

## Methods

### Study design

For the present article, we retrospectively analysed data that we collected for a study which assessed the efficacy of a 14-week progressive HIIT protocol for nondiabetic, premenopausal previously-sedentary AA women with overweight/obesity. The results of that study have been published previously [21]. For that study, training sessions took place in the exercise physiology laboratory at Mount Sinai Morningside (formerly known as Mount Sinai St. Luke’s) Hospital. All experimental procedures were submitted to and approved by the St. Luke’s Roosevelt Institute for Health Science Institutional Review Board. This study adheres to the standard checklist of CONSORT, which provides guidelines for the reporting of clinical trials.

### Participants

Twenty-eight healthy, premenopausal (age, 20–40 yr), sedentary (exercise frequency/duration, < 3 times/wk., 60 min/session), nondiabetic (fasting blood glucose, < 110 mg∙dl^− 1^), overweight/obese (BMI, > 25 kg∙m^− 2^) AA women volunteered to participate in this randomized controlled clinical trial. All participants gave their written informed consent prior to commencement of the study after procedures, associated risks and potential benefits of participation had been explained. Being that the training protocol required visiting the laboratory three times per week for 14 weeks, we focused our recruitment efforts on the area in proximity to the hospital where the laboratory was located (East Harlem), a predominantly low-income, working-class minority community with higher than average unemployment and poverty rates [23]. Consequently, although not part of the methodological design of the study, it is intuitive that the monetary incentive we provided might have been particularly conducive to improving adherence for the participants that we recruited. Participants were considered for inclusion only if they self-reported that all four grandparents were AA. Exclusion criteria included: 1.) weight change > ± 2 kg within the past 3 mo; 2.) medication intake that might affect insulin or fat metabolism (including oral contraceptives); 3.) smoking within the past 6 mo; 4.) consumption of > 2 oz. ethanol per day; and/or 5.) having irregular menstrual cycles (e.g., skipping > 2 monthly cycles per year). Individuals who were deemed eligible for participation based on these criteria then underwent a full physical examination, which included blood work, resting ECG, and oral glucose tolerance test (OGTT) to ensure absence of diabetes (2-h OGTT plasma glucose, < 140 mg∙dl^− 1^), hyperlipidemia (fasting plasma triglycerides, < 350 mg∙dl^− 1^ and total cholesterol < 300 mg∙dl^− 1^) and other chronic illnesses that might affect their capacity to exercise. After this assessment, without stratification, participants were randomly allocated to a HIIT training group (*n* = 14) or a no-exercise control group. The data which were retrospectively analysed for this article represent those which were collected for the individuals in the HIIT group who completed the 14-week intervention (*n* = 9; age, 29 ± 4 yr; body mass, 90.1 ± 13.8 kg; BMI, 32.4 ± 3.6 kg∙m^− 2^; overweight/obese, *n* = 3/6). Participants received $300 for completing pre and post measurements and $50 per week during the 14-week intervention period. For more detailed information about the participants who were assessed in this study, please see the parent study from which these data were extracted [21].

### Procedure

Members of both groups performed a step-incremental cycling test for determination of the peak rate of oxygen uptake (V̇o_2peak_), gas exchange threshold (GET) and limit of exercise tolerance (T_lim_) prior to and following the 14-week intervention period of the study. However, participants did not perform an additional constant-work-rate severe-intensity cycling bout following the incremental test to ‘verify’ V̇o_2max_ nor were ‘secondary criteria’ assessed due to concerns about their validity [24, 25]. For the HIIT group, the intervention consisted of 24-min HIIT exercise sessions performed three times per week for 13–15 weeks depending on timing of the participant’s menstrual cycle (pre- and post-intervention testing was done during the follicular phase). The training period consisted of four progressive stages during which both work-interval intensity and work/recovery ratio were progressed (see Table 1). An exercise physiologist supervised all exercise sessions with intensity prescribed, monitored and recorded as a percentage of heart-rate reserve (HRR), which was determined from data collected during the pre-intervention incremental test. The exercise training was performed on a cycle ergometer (Monark, 927E, Hasbro, Sweden). Each session began with 6 min of warm-up cycling at 50% HRR after which four work intervals were performed with recovery intervals at 50% HRR interspersed. In an attempt to achieve/maintain the target HR during each interval, the test supervisor adjusted the load against which the participant was cycling and/or instructed the participant to adjust their pedal cadence accordingly. Specifically, if the HR was below the target HR for that stage (see Table 1), the test supervisor increased the load if cadence was within the desired range (50–70 rpm) or instructed the participant to pedal faster. The test supervisor also recorded participant rating of perceived exertion (RPE) before and after each work interval. A ‘cool-down’ comprising 5 min of low-intensity cycling was performed following completion of the final work interval. Participant body mass was monitored and dietary counselling was provided by a registered dietitian to ensure weight stability (i.e., prevent weight loss that might occur when the addition of exercise to a sedentary individual’s regimen creates a negative energy balance compared to their normal hyper- or iso-energetic intake) throughout the 14-week intervention. Sample-size calculations were performed for the primary aims of the parent study based on power = 0.80 for two-tailed α = 0.05 with Cohen’s *d* for effect size (0.84) using preliminary data that we collected and data that were presented in previously-published articles [26–28]. For more detailed information about the testing and training protocol employed in this study, please see the parent study from which these data were extracted [21].

**Table 1:** Intensity and work/recovery characteristics for the 14-week HIIT exercise intervention which was performed three times per week by previously-sedentary overweight/obese AA women in this study

Week	Intensity (% HRR)	Work Interval (s)	Recovery Interval (s)	Work/Recovery Ratio
**Stage 1**				
1	75	30	210	1.0/7.0
2	75	45	195	1.0/4.3
3	75	60	180	1.0/3.0
**Stage 2**				
4	80	60	180	1.0/3.0
5	80	60	180	1.0/3.0
**Stage 3**				
6	85	60	180	1.0/3.0
7	85	60	180	1.0/3.0
**Stage 4**				
8	90	60	180	1.0/3.0
9	90	60	180	1.0/3.0
10	90	60	180	
11	90	60	180	1.0/3.0
12	90	60	180	1.0/3.0
13	90	60	180	1.0/3.0
14	90	60	180	1.0/3.0

### Data analysis

The feasibility of this progressive HIIT exercise program was assessed based on adherence (attrition rate), participant perception (RPE) and success rate, which was calculated based on the degree to which target-intensity values for the work intervals that comprised the four progressive stages (see Table 1) could be achieved/maintained. Specifically, the test supervisor recorded the number of intervals during which the target HR was achieved/surpassed for the four intervals performed during each workout for each participant. This allowed us to calculate the percentage of successful intervals for each stage of the study.

### Statistical analysis

The percentage of work intervals for which the target HR was achieved/surpassed was compared across the four stages of the 14-week intervention using a one-way repeated-measures ANOVA with LSD post-hoc analysis. The RPE values for the four stages were compared in a similar manner. Pearson’s correlation coefficients were used to investigate the relationship between participant characteristics at baseline (age, body mass, BMI and V̇o_2peak_ expressed relative to body mass and fat-free mass) and the ability to maintain the target intensity across the four progressive stages, which was quantified by determining the percent change in success rate between stage 1 and 4 (∆SR_4–1_). Pearson’s correlation coefficients were also used to assess the relationship between the change in RPE between stage 1 and 4 (∆RPE_4–1_) and ∆SR_4–1_. In all cases, statistical significance was accepted when *p* < 0.05.

## Results

### Adherence

Five of 14 participants (35%) in the HIIT group dropped out during the 14-week intervention. Three of these dropouts occurred during the first 5 weeks of training (i.e., during training at the lower range of the intensity spectrum; 75–80% HRR); specifically, two during week 3 (session 7 and session 8) and one during week 4 (session 11). However, two of these three dropouts occurred due to the inconvenience caused by Hurricane Sandy (October 2012) as opposed to lack of tolerance of the training protocol per se. The other participant who dropped out during the initial two stages of training did so citing an inability to satisfy the time commitment associated with attending the exercise sessions and both of the other dropouts occurred for the same reason: one during week 7 (session 20; 85% HRR) and one during week 11 (session 32; 90% HRR). Three of 14 participants in the control group (21%) dropped out prior to post-intervention testing. Reasons for these dropouts were dissatisfaction with the no-exercise/weight maintenance requirements of the control group (*n* = 2) and relocation (*n* = 1). One of the nine participants who completed the HIIT intervention did not follow the program as prescribed during stage 3 (inconsistent attendance); hence, her data are not included in the analyses that involved all four stages.

### Participant perception

The group mean ± SD for the RPE registered upon completion of the pre- and post-training incremental test by seven of the nine members of HIIT (an RPE measurement was not recorded for the post-training test for two participants) were 17.9 ± 1.9 and 18.6 ± 2.0, respectively. The group mean ± SD for the RPE registered upon completion of the work intervals performed during each of the four stages of the intervention are presented in Table 2. There was no significant difference in RPE across the four stages; however, a ‘trend’ (*p* = 0.057) was present with post-hoc analysis revealing differences between stage 1 and 2 and stage 1 and 3 (*p* = 0.018 and 0.005, respectively).

**Table 2:** The group mean ± SD for perceptual responses as indicated by RPE measurement (Borg; 6–20) that were conducted following each of the work intervals performed averaged for the four stages of progression in this study

Stage	Average for	RPE
1	Weeks 1–3	16.0 ± 2.0
2	Weeks 4–5	16.9 ± 2.3
3	Weeks 6–7	17.6 ± 1.9
4	Weeks 8–14	17.4 ± 1.2

### Success rate

**Fig. 1:**
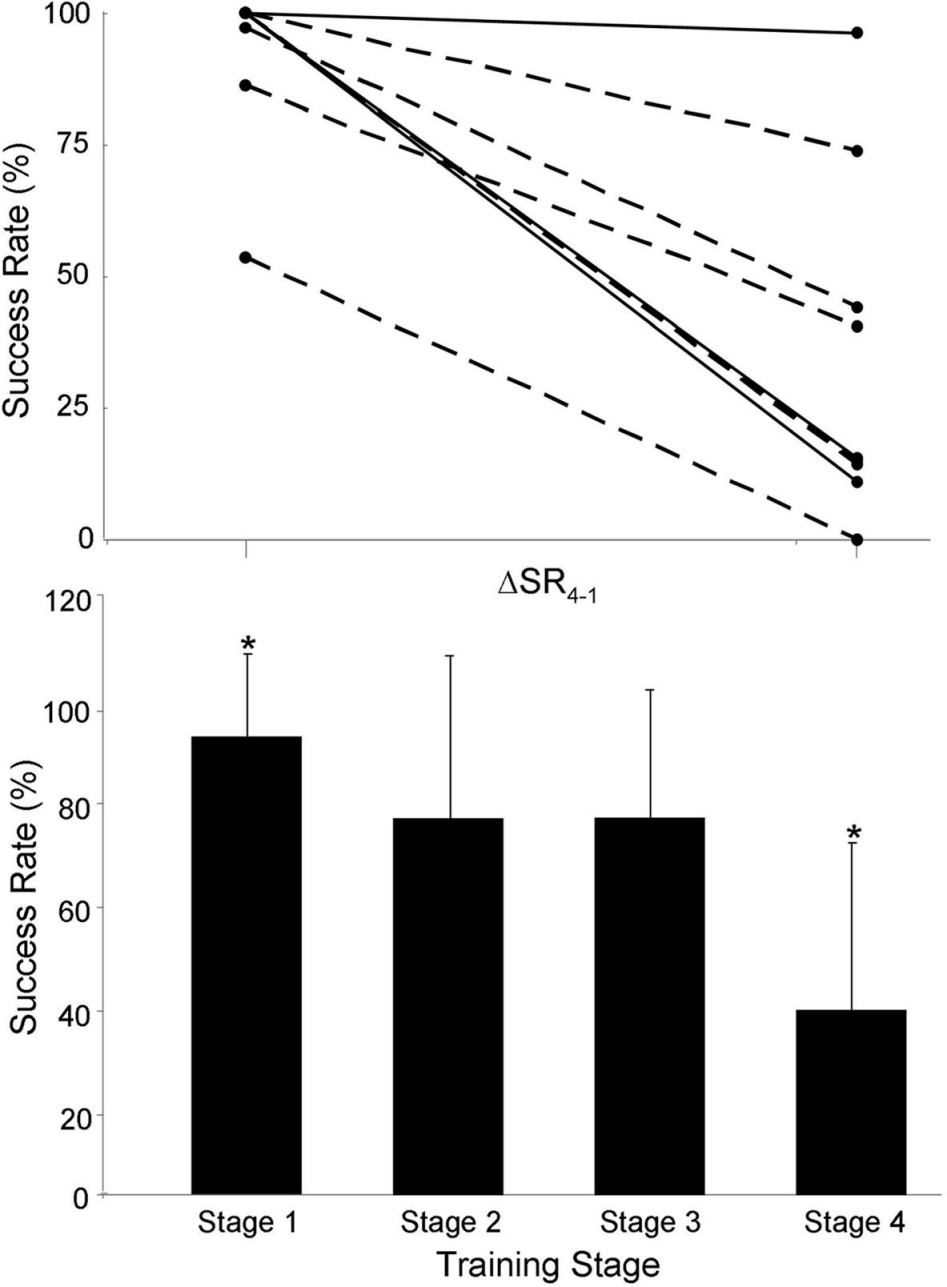
Top panel depicts individual-participant data (overweight, solid line; obese, dashed line) for the difference in success rate between stage 1 and 4 (∆SR_4–1_). Bottom panel depicts the group mean ± SD for success rate for each of the four stages of the 14-week HIIT intervention. * *p* < 0.05 compared to other three stages

**Fig. 2:**
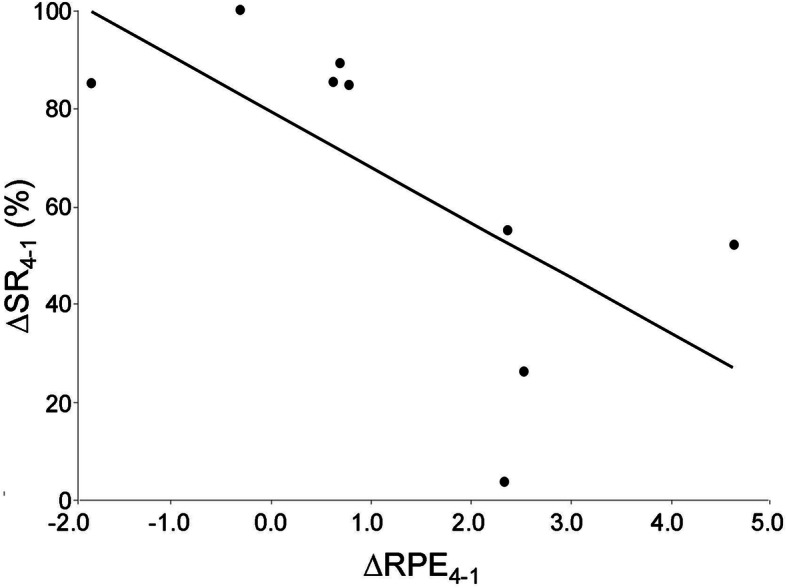
Inverse relationship (*p* = 0.058) between the difference in RPE between stage 1 and 4 and the difference in corresponding success rate (∆RPE_4–1_ and ∆SR_4–1_, respectively)

The group mean ± SD for the percentage of work intervals for which the target HR was achieved/surpassed for participants who completed the intervention is depicted in the lower panel of Fig. 1. A significant difference was detected across stages (*p* = 0.018) with post-hoc analysis revealing that stage 1 was characterised by a significantly greater value compared to the other three stages while stage 4 was characterised by a significantly reduced value compared to the other three stages. There was no significant difference between stages 2 and 3. Individual-participant data for the difference between stage 1 and 4 is depicted in the top panel of Fig. 1. There was no significant correlation between any of the participant baseline characteristics (see above) and ∆SR_4–1_ whereas a ‘trend’ for an inverse relationship was observed between ∆RPE_4–1_ and ∆SR_4–1_ (*r* = − 0.651; *p* = 0.058) (see Fig. 2).

## Discussion

The main finding from this investigation is that a progressive HIIT program involving work intervals at intensities which were increased from 75 to 90% HRR over a 14-week intervention period might be too aggressive for overweight/obese AA women that were previously sedentary. Specifically, our experimental hypothesis was refuted because a significant reduction in the percentage of successful work-interval sessions was observed for the three stages of progression following stage 1 (75% HRR for the first 3 weeks) with a precipitous drop for stage 4 (90% HRR for the final 7 weeks; see Fig. 1). However, considering our small sample size, this should be considered only as a preliminary finding with confirmation that such intensity progression is not feasible for this subject population requiring future studies with more participants. With similar caution due to the limited number of participants we recruited for our training group (*n* = 14), we found a 35% attrition rate that was higher than that which is typically found when HIIT is performed by previously-sedentary individuals in general (~ 17.6%) [12], but in line with that which has been reported when it is performed by previously-sedentary overweight/obese AA women [22]. Collectively, we interpret these findings to imply that a 14-week progressive HIIT intervention comprising three sessions per week might be better tolerated by overweight/obese previously-sedentary AA women if work-interval intensity is restricted to < 90% HRR. However, in addition to sample size, more research is required to confirm that this is the case when the program is implemented in a ‘real-world’ setting with the objective of long-term participation without the monetary incentive we provided.

African American women possess lower cardiorespiratory fitness [14, 15] and a higher prevalence of obesity [16] and insulin resistance [17] compared to their white counterparts and also tend to be less physically active [13]. To counteract the metabolic repercussions of these differences, lifestyle changes including caloric restriction and increased physical activity have been recommended [13]. With respect to the latter, developing appropriate exercise interventions for AA women is, therefore, important; however, an attempt to do so is confounded by a number of barriers that these women face that have been implicated in their vulnerability to the physically-inactive lifestyle [29]. One of these barriers is lack of time [13, 29] which is why time-efficient HIIT might be a particularly attractive option for this group. For example, in the present study, the actual HIIT session including warm-up and cool-down required only ~ 40 min to complete. However, three participants still cited an inability to satisfy the time commitment associated with attending the exercise sessions as their reason for dropping out despite the fact that, generally speaking, participants lived in proximity to where training sessions were conducted, which was a hospital that was readily accessible via public transportation. This implies that at least for this type of participant, in some cases, even the reduced time commitment required for HIIT exceeds that which they are willing to expend.

We are aware of only one other study that has investigated HIIT for AA women: Hornbuckle et al. found that HIIT on a treadmill was more effective at reducing waist circumference and increasing the frequency of steps per day than continuous treadmill walking at a sustained pace for young previously-sedentary overweight/obese AA women [22]. Interestingly, of the 27 women recruited for that study, only 14 (52%) completed the 16-week intervention; however, eight out of 11 dropouts occurred in the continuous-exercise group. Importantly, this dropout rate was significantly greater than what they observed for the HIIT group (31%), which was in line with what we found in retrospective analysis of our data (35%). Furthermore, two of the five dropouts in our study occurred due to the inconvenience caused by Hurricane Sandy, which was an extraordinary event that would have likely reduced adherence regardless of cultural influence. However, previous research predicts that our protocol, which involved non-weight-bearing stationary cycling, should have resulted in better adherence compared to treadmill training [12]. Furthermore, it is important to note that both our participants and those of Hornbuckle et al. received monetary compensation in exchange for their participation, which starkly contrasts the 'real world' where a monetary outlay is often required to incorporate exercise (particularly that of a high-intensity nature) into the daily regimen. Considering the demographic composition of the area from which we recruited [23], it is likely that our participants were of a lower socioeconomic status, which makes this of even greater concern. Nevertheless, given the high rate of attrition that has been reported upon initiation of training for sedentary individuals per se [30], the lower dropout rate that has been observed when previously-sedentary individuals perform HIIT compared to traditional training [12] and the challenges that face sedentary AA women in particular [29], it is attractive to speculate that our findings [21] and those of Hornbuckle et al. [22] regarding HIIT for previously-sedentary overweight/obese AA women are promising and require further investigation.

Albeit based on a small sample size for individuals who completed our 14-week protocol (*n* = 9), in addition to information regarding exercise adherence, retrospective analysis of our data suggests that the progressive protocol we employed might be too aggressive to be sustained over the course of the intervention. Specifically, a drop in the ability to satisfy the target intensity during HIIT work intervals occurred following the initial stage of training with a subsequent precipitous drop when attempting the requirements of stage 4 (Fig. 1). In this regard, it is important to recognise that there is considerable latitude when establishing program variables for protocols that fall under the HIIT umbrella term. For example, in the aforementioned study on AA women by Hornbuckle et al., these researchers chose to have participants perform a HIIT protocol (weeks 5–16) which comprised seven 60-s work intervals at 80–90% of the maximum HR (HR_max_) separated by 180 s of recovery at 60–70%. Given the different way of intensity quantification compared to our method (percent of HR_max_ *v.* percent of HRR), work intervals throughout the entirety of that study were likely performed in the range of target intensities that we used during the first stage of training (i.e., weeks 1–3; 75% HRR ≈ 87% HR_max_) [21, 22]. Moreover, the fact that it is ‘easier’ to elicit a given target HR during treadmill (i.e., ‘full-body’) exercise compared to leg cycling is another difference between studies that deserves mention. Nevertheless, differences aside, collectively, the data from these two studies appear to indicate that target intensities at the lower end of the HIIT intensity range are most appropriate when 60-s work intervals are performed during HIIT training for overweight/obese AA women during the initial stage of training. Interestingly, instead of progressing intensity like we did, Hornbuckle chose to progress from an ‘adaptation phase’ comprising continuous training (weeks 1–4) to the HIIT protocol [22] compared to starting the protocol with HIIT. In retrospect, this might be a more feasible way to allow for progression during the initial stage of training for this type of individual compared to the method we employed. However, it is also important to recognise that while significant, the reduction in success rate between stage 1 and stage 2/3 in our study was moderate compared to the large drop which occurred for stage 4 (see Fig. 1). With this in mind, in addition to an adaptation period (e.g., weeks 1–4) from continuous to interval training, it might be feasible for progression from 75 to 80–85% HRR in a more protracted manner than we employed over the course of the following 10 weeks. Future research should be designed to explore this possibility.

Although beyond the scope of the present analysis, it is interesting to speculate on why participants were unable to successfully span the progressive range of intensity (and, specifically, achieve the 90%-HRR target) that we prescribed. Generally speaking, by observation, the test administrator believed it to be lack of motivation because individuals who were successful (see top panel of Fig. 1) appeared to be working more vigorously than those who were not. Specifically, those who were unable to achieve the target intensity appeared unwilling to tolerate the requisite degree of effort despite the administrator’s strong encouragement. The trend for an inverse relationship between ∆SR_4–1_ and ∆RPE_4–1_ (Fig. 2), a parameter that should reflect how much participants were willing to progress enduring the feeling of physical exertion as they were asked to progress exercise intensity over the 14 weeks, is consistent with this observation.

While we hope that the findings we have presented will provide preliminary data to inform future research, in addition to our small sample size (see above), there are a number of other limitations that must be acknowledged. While we did use the well-accepted Borg scale to measure the perceptive response during exercise (i.e., the perceived degree of physical exertion that the exerciser was experiencing), we did not assess affective response. Consequently, while we know *what* participants were feeling while they were exercising, we do not know *how* they were feeling [31]. Importantly, Rose and Parfitt suggest that regulating exercise intensity by affective state is advantageous compared to monitoring HR or RPE because a positive affective response will provide greater motivation to repeat the experience in the future [32]. In support of this contention, while finding some commonality in the variance of future behaviour that was explained by both affective response and RPE, Williams et al. confirmed that the ability of the former to predict physical-activity participation at six and 12 months was mostly independent of the latter [33]. With respect to long-term adherence, monitoring affective response in addition to HR and RPE might, therefore, be a more comprehensive approach when determining the feasibility of an initial HIIT intervention. It is also important to note that the progressive-overload scheme that we employed (i.e., an increase from 75 to 90% HRR over the course of a 14-week initial intervention) was chosen somewhat arbitrarily based on general guidelines that have been advanced for HIIT [3] as opposed to definitive tenets which, to the best of our knowledge, have yet to be established. However, for this type of individual, compared to volume, progression based on increasing intensity appears to be the better way to improve prognosis via exercise training. For example, Ramos et al. found that when performed three times per week, a HIIT intervention reduced disease severity for individuals diagnosed with metabolic syndrome to the same extent regardless of whether it consisted of 4-min work intervals at 85–95% of the maximum HR (RPE of 15–17) repeated once or four times [34]. The fact that the benefit of an initial 16-week HIIT protocol was not enhanced by an increase in volume (i.e., weekly accumulation of 114 min of total exercise compared to 51) implies that intensity progression through the typically-recommended range is the most viable way to progress training load at least during the initial stages of HIIT [34]. However, unlike the present study, Ramos et al. did not compare different intensities of HIIT and, specifically, an intensity range that might be unattainable for the type of individual that we assessed. Hence, their findings simply indicate a threshold volume of HIIT that is required to induce beneficial effects for individuals with metabolic syndrome in general. The fact that higher training volume is associated with an increased dropout rate for sedentary individuals performing HIIT [12] also supports use of intensity progression during an initial HIIT intervention with the caveat that such progression must be constrained to a feasible range.

## Conclusions

With the caveat that we draw this conclusion based on a limited number of participants who completed the protocol, a HIIT intervention comprising three sessions per week appears to be better tolerated by previously-sedentary overweight/obese AA women if work-interval intensity is restricted to < 90% HRR throughout the initial 14 weeks of training. Progression from a beginning phase of continuous training and progression to intensities ranging from 80 to 85% HRR during the latter stages of such an initial intervention are areas of future research that should be explored as is the feasibility of this type of program for previously-sedentary overweight/obese AA women in a real-world setting without monetary incentive being provided.

## Data Availability

The data collected during this investigation are available from the corresponding author upon request.

## Abbreviations

AA: African American
BMI: Body mass index
GET: Gas exchange threshold
HIIT: High-intensity interval training
HR: Heart rate
HR_max_: Maximum heart rate
HRR: Heart rate reserve
RPE: Rating of perceived exertion
T_lim_: Limit of exercise tolerance
V̇o_2peak_: Peak rate of oxygen uptake
